# Natural products as new antimitotic compounds for anticancer drug development

**DOI:** 10.6061/clinics/2018/e813s

**Published:** 2018-11-27

**Authors:** Carlos Roberto Koscky Paier, Sarah Sant'Anna Maranhão, Teiliane Rodrigues Carneiro, Lídia Moreira Lima, Danilo Damasceno Rocha, Renan da Silva Santos, Kaio Moraes de Farias, Manoel Odorico de Moraes-Filho, Claudia Pessoa

**Affiliations:** I Laboratorio de Oncologia Experimental, Nucleo de Pesquisa e Desenvolvimento de Medicamentos (NPDM), Universidade Federal do Ceara, Fortaleza, CE, BR; II Programa de Pos graduacao em Farmacologia, Universidade Federal do Ceara, Fortaleza, CE, BR; III Programa de Pos graduacao em Biotecnologia, Rede Nordeste de Biotecnologia (RENORBIO), Universidade Federal do Ceara, Fortaleza, CE, BR; IV Laboratorio de Avaliacao e Sintese de Substancias Bioativas (LASSBio), Instituto de Ciencia e Tecnologia de Farmacos e Medicamentos (INCT-INOFAR), Universidade Federal do Rio de Janeiro, Rio de Janeiro, RJ, BR

**Keywords:** Antimitotic Agents, Microtubules, Spindle Apparatus, Mitosis, Cancer

## Abstract

Cell cycle control genes are frequently mutated in cancer cells, which usually display higher rates of proliferation than normal cells. Dysregulated mitosis leads to genomic instability, which contributes to tumor progression and aggressiveness. Many drugs that disrupt mitosis have been studied because they induce cell cycle arrest and tumor cell death. These antitumor compounds are referred to as antimitotics. Vinca alkaloids and taxanes are natural products that target microtubules and inhibit mitosis, and their derivatives are among the most commonly used drugs in cancer therapy worldwide. However, severe adverse effects such as neuropathies are frequently observed during treatment with microtubule-targeting agents. Many efforts have been directed at developing improved antimitotics with increased specificity and decreased likelihood of inducing side effects. These new drugs generally target specific components of mitotic regulation that are mainly or exclusively expressed during cell division, such as kinases, motor proteins and multiprotein complexes. Such small molecules are now in preclinical studies and clinical trials, and many are products or derivatives from natural sources. In this review, we focused on the most promising targets for the development of antimitotics and discussed the advantages and disadvantages of these targets. We also highlighted the novel natural antimitotic agents under investigation by our research group, including combretastatins, withanolides and pterocarpans, which show the potential to circumvent the main issues in antimitotic therapy.

## INTRODUCTION

Cancer pathogenesis is a complex process in which normal cycling cells acquire basic neoplastic characteristics typically common to all tumor types. Cells from normal tissues exhibit growth entirely controlled by the cell division machinery, which is orchestrated by the production, release, recognition and inhibition of growth-promoting signals to foster tissue homeostasis. Tumor cells escape from this growth orchestration through various pathways, usually those linked to self-sufficient growth. Therefore, an essential feature of cancer cells is the ability to maintain chronically disrupted homeostatic proliferation 1. Mitosis is the fundamental proliferation process of division in human somatic cells; dysregulated mitosis leads to genomic instability characterized by DNA mutations and chromosomal aberrations. These genetic changes result in proliferative advantages for neoplastic cells and increase the susceptibility of these cells to the accumulation of additional genetic mutations, which in turn contribute to tumor progression and an exacerbated aggressive phenotype 2.

**Figure 1:**
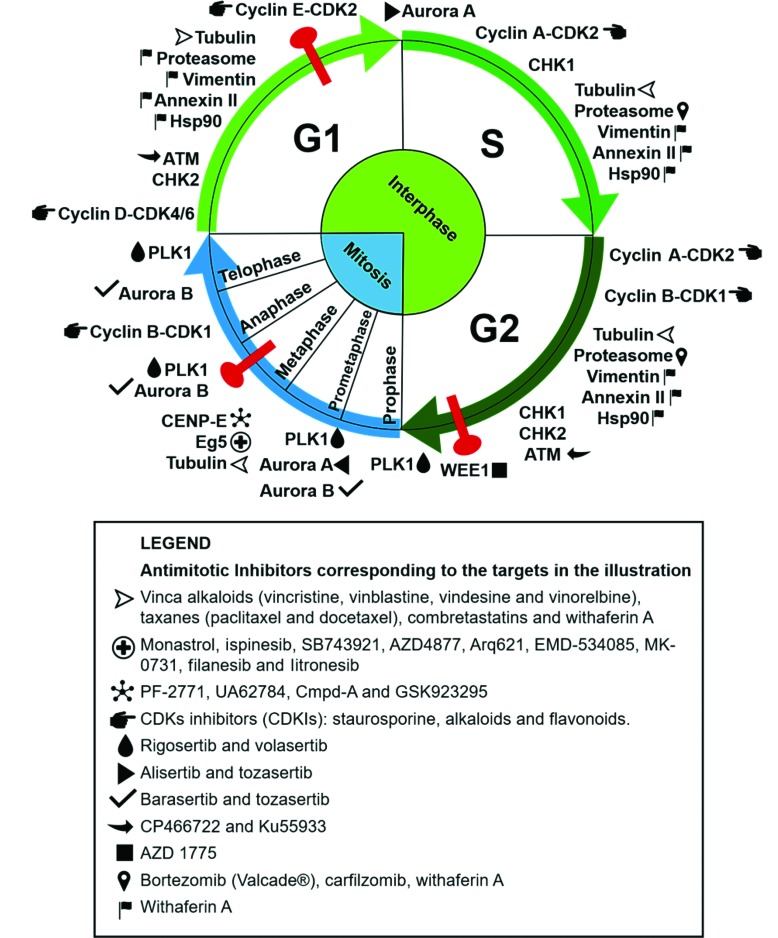
Antimitotic targets and their respective inhibitors as cited in the text. The targets are placed in the illustration according to the cell cycle phase in which they perform their main functions. The corresponding inhibitors are listed in the legend. The phases of mitosis are depicted in blue, the phases of interphase are in green, and cell cycle checkpoints are in red. The compounds (+)2,3,9-trimethoxypterocarpan and withaphysalin F are not included, because their targets are unknown.

Some compounds used in cancer chemotherapy target protein components of the mitotic regulatory machinery, acting as cell division blockers and, consequently, cell death inducers 3. Many proteins involved in this process play an essential role in mitosis, making them interesting targets for the development of antimitotic anticancer drugs. The clinical success of compounds that disrupt microtubule dynamics, such as vinca alkaloids and taxanes, stimulated the development of new drugs with specific mitotic targets 4. Substantial research aimed at the development of new antimitotic compounds with different targets and increasingly selective antitumor activity has ensued. Herein, we discuss traditional and novel antimitotics with antitumor potential and the respective cellular targets of these agents, as summarized in Figure 1.

### Antimitotic agents and their main targets

Mitosis is a crucial phase of the cell cycle when the chromosomes must be aligned on the metaphase plate and segregated correctly to generate two identical daughter cells. The search for new clinically effective antitumor compounds has yielded many drugs directed at targets expressed during mitosis. Such compounds, called antimitotics, target proteins involved in this complex process and are mitotic arrest agents 5.

### Microtubules and microtubule-associated proteins

Microtubules are composed of α- and β-tubulin heterodimers that rapidly polymerize and depolymerize. Many microtubule-associated proteins (MAPs) fine-tune the organization of microtubules and promote or suppress microtubule dynamics 6. Microtubules play important roles in a wide range of cellular functions, such as the assembly of the mitotic spindle; the movement of organelles, vesicles and proteins; and the associated cell signaling, as well as in the development and maintenance of cellular shape 7. Chromosome segregation is first enabled by the connection between mitotic spindle microtubules and the kinetochore, a macromolecular complex positioned at the chromosomal centromere, which is assembled at the beginning of mitosis 4. Coupling between microtubules and the kinetochore is essential for correct spindle formation and subsequent chromosome alignment and segregation 6. Unsurprisingly, the first antimitotic drugs developed interfere with microtubule dynamics, through either the inhibition or stabilization of microtubule polymerization.

Tubulin-targeting antimitotics bind tubulin at different sites and may exert different effects. Vinca alkaloids (vincristine, vinblastine, vindesine, vinorelbine and vinflunine) are natural products capable of binding and destabilizing the polymeric structure of microtubules, thus causing microtubule disassembly. Colchicine also acts via this mechanism but at another interaction site. Taxanes (paclitaxel and docetaxel) and epothilones are examples of microtubule-stabilizing agents that prevent depolymerization 8. Tubulin-binding agents traditionally used in the clinic kill dividing tumor and healthy cells and affect microtubule dynamics in nonproliferating cells, resulting in various side effects, such as myelosuppression and neuropathies 9. Nevertheless, tubulin-binding agents are widely employed in the treatment of several solid tumors and oncohematological malignancies 8.

After the first tubulin-binding agents showed antitumor effects, many other tubulin-targeting antimitotic drugs were developed, along with related inhibitors directed at other targets such as kinases and mitotic protein complexes, aimed at inhibiting cell proliferation 5. Among the MAPs, the kinesin spindle protein (KSP) Eg5 is a kinesin responsible for the separation of centrosomes and for the bipolar configuration of the mitotic spindle. Inhibition of Eg5 causes monopolar spindle formation and cell cycle arrest, an effect demonstrated by monastrol, a small-molecule Eg5 inhibitor 10,11. Other Eg5 inhibitors, such as ispinesib (Cytokinetics), SB743921 (Cytokinetics), AZD4877 (AstraZeneca), Arq621 (ArQule), EMD-534085 (Merck-K GaA), MK-0731 (Merck & Co.), filanesib (Array Biopharma) and litronesib (Kyowa Hakko Kirin/Eli Lilly), have reached clinical trials 12. Centromeric protein E (CENP-E) is another MAP inhibited by antitumor agents. CENP-E is essential for the alignment of chromosomes during metaphase and passage to anaphase 3. Examples of CENP-E inhibitors are PF-2771 13, UA62784 14 and Cmpd-A 15, but only GSK923295 has reached clinical trials 16. Abnormalities in the function and expression of kinesins are important for the development and progression of many human cancers, suggesting that this class of proteins is an interesting target for new anticancer therapy strategies 17,18. Since some kinesins are expressed only during mitosis, their inhibition may reduce side effects related to antineoplastic agents targeting interphase proteins, such as traditional tubulintargeting antimitotics 9.

### Cell cycle regulatory kinases

Mitosis is a strictly controlled set of events driven by many regulatory proteins that ensure cellular modifications suitable for the correct replication and distribution of genomic DNA between daughter cells. Among the proteins targeted by new antitumor agent prototypes are cell cycle regulators such as checkpoint kinases (CHKs) and cyclin-dependent kinases (CDKs), as well as regulators of the mitotic process, including Aurora kinases (AURKs) and polo-like kinases (PLKs) 19.

CDKs and CHKs control cell cycle progression. CHKs are responsible for maintaining cell genomic integrity since they are activated in response to DNA damage detected by molecular checking mechanisms in a temporally and spatially controlled manner. Activation of CHK1 and CHK2 results in cell cycle arrest, so that DNA is repaired or, if repair is not possible, apoptosis can be triggered. The defects in checkpoint signaling in many types of cancer are important drivers for the development of new drugs targeting CHKs 20. In addition, CDKs are kinases that control crucial steps in the cell cycle and are regulated by binding proteins called cyclins. Gene products that promote cell cycle progression are often mutated and constitutively active in tumor cells, increasing the interest in the development of selective inhibitors of CDKs and mitotic kinases 21. CDK inhibitors (CDKIs) include staurosporine, alkaloids and flavonoids, many of which have undergone clinical studies 22,23.

PLK1 is the most studied member of the PLK family, with important roles in some cell cycle steps, such as the activation of the CDK1-cyclin B complex, the onset of chromosomal segregation and cytokinesis 25. The expression of PLK1 is often elevated in tumors, which is correlated with a poor prognosis; thus, PLK1 is a target for anticancer therapy 26. Indeed, the development of PLK inhibitors is currently focused on PLK1 and has resulted in two promising inhibitors in clinical trials, namely, rigosertib and volasertib 27,28.

The Aurora family consists of serine-threonine kinases with a pivotal role in the control of the cell cycle and mitosis. Aurora A is located in the centrosome and acts in the progression from the G1 phase to the S phase of the cell cycle and in the initial stages of mitosis, enabling centrosome separation and mitotic spindle assembly. Aurora B is found in the mitotic spindle and controls chromosome condensation, in addition to acting at the end of mitosis to regulate cytokinesis 29. Both Auroras have an oncogenic role in human tumors 30-33. Among the effects associated with tumorigenesis, Aurora A overexpression leads to chromosomal instability characterized by aneuploidy and the premature segregation of sister chromatids 34. Inhibition of Aurora kinases is a strategy in cancer treatment, especially in combined therapeutic approaches. Most Aurora inhibitors, such as alisertib, tozasertib and barasertib, compete with ATP for binding to the catalytic site 35-37.

### Nonmitotic targets affecting the cell proliferation rate

Functional interphase proteins are targets that disrupt the cell cycle and prevent mitosis via DNA repair modulation, protein turnover and checkpoint signaling. Targeting these nonmitotic proteins may hinder cell division. For example, the ataxia-telangiectasia mutated (ATM) kinase induces a cell signaling cascade in response to DNA double-strand breaks. When activated, ATM phosphorylates substrates that promote DNA repair and, consequently, cell cycle progression 38. Somatic mutations in the ATM gene are found in several cancers, especially in hematological malignancies, and are often associated with chemotherapy resistance and unfavorable prognoses 39,40. Due to the substantial influence of the ATM kinase on cell cycle progression, the development of ATM-specific inhibitors is a good strategy in the search for new anticancer therapies 41. For example, the ATM inhibitors CP466722 and Ku55933 were identified through a cell-based high-throughput screening assay 38.

WEE1 protein is a major tyrosine kinase involved in the progression of the cell cycle beyond the checkpoint between the G2 phase and mitosis (M phase), preventing entry into the mitotic cell division process in response to persistent DNA damage. High expression of this protein is often found in cancers (colorectal cancers, breast cancers, leukemias and other cancers) and correlates with tumor progression and negative prognoses, despite the cell cycle-arresting activity of WEE1 before mitosis onset 42-44. This apparent contradiction is not yet fully understood, and the current hypothesis is that WEE1 inhibition would promote the entry of cells with excessive DNA damage into mitosis, which would induce cell death during the ensuing M phase. WEE1 kinase inhibitors, e.g., AZD1775, have been studied in clinical trials, but their use is limited because of their nonselective action 45-47.

The 26S proteasome is an important multimeric complex responsible for the degradation of misfolded or overexpressed proteins 48. Neoplastic cells exhibit high dependence on the 26S proteasome because of the need to eliminate defective proteins, which are detrimental to cell cycle advancement, resulting from the genetic instability in the tumor and the high cell proliferation rate. The accumulation of ubiquitinated and misfolded proteins may induce apoptosis. Therefore, the 26S proteasome is a validated therapeutic target in oncology, with FDA-approved inhibitors such as bortezomib (Velcade^®^) and carfilzomib currently in clinical use 49 for the treatment of multiple myeloma, in which malignant cells have an abundance of proteasomes 49,50.

In addition to the proteins cited above, many others may be validated as targets for cancer therapy. A better understanding of mitotic mediators and their role in tumorigenesis may advance the fight against highly proliferative tumor cells.

### Challenges in the search for new mitotic targets for anticancer therapy

Many efforts have been made in the search for and development of new antitumor compounds; however, cancer remains a difficult-to-treat disease 51. The available antimitotic therapies are somewhat successful but rarely eradicate tumors completely and generally promote tumor resistance and systemic toxicity 52. The selectivity of antimitotics for tumors has long been believed to be based on the high proliferation rate of cancer cells in contrast to the supposed quiescence or low division rate of most cells in normal tissues. Normal and tumor cells progress through the cell cycle in similar ways, but the higher proliferation rates of cancer cells cause mitotic targets to be more frequently expressed in tumors than in normal tissues, thus making neoplastic cells more sensitive to antimitotic action 52. However, Komlodi-Pasztor and colleagues emphasized that tumor proliferation rates may not be as high as expected, and thus the frequency of mitotic target expression may not be as different as expected between tumor and nontumor cell populations. This characteristic could be a reason for the lack of specificity of antimitotic agents such as mitosis-specific kinase inhibitors 53.

In addition to selectivity issues, antimitotic drugs acting only on M phase-exclusive targets allow interphase cancer cells to be refractory to treatment. In his review, Mitchison 54 compared data from the literature and showed that 2-5% of cells in primary tumors are in the S phase, while 40% of cells in *in vitro* tumor cell cultures are in the S phase. In addition, the doubling times of tumor cells in patients are very long, approximately fifty times greater than those in cell lines or animal models. Furthermore, the cell proliferation ratio varies greatly according to different cancer types, individual patient characteristics, and the primary tumor origin and location. Therefore, the number of neoplastic cells in interphase is probably high enough to make a tumor resistant to chemotherapeutics that act specifically on exclusive mitotic targets. This fact may explain the preclinical failure of many antimitotic compounds 52,55.

Antimitotics are considered mitosis blockers and, consequently, cell death inducers. The main issue in antimitotic therapy is that not all cells blocked in M phase will die, with many becoming aneuploid. This alteration is a direct consequence of chromosomal instability, that is, susceptibility to the gain and/or loss of chromosomes, which generates an unstable karyotype. This instability promotes the acquisition of malignant tumor characteristics as a proliferative advantage due to the increased mutation rate in oncogenes and tumor suppressor genes 55. The gain of malignant features by tumors hinders the prediction of patient response to therapy and facilitates the emergence of chemotherapy-resistant tumor cells. Treatment resistance is a major problem that hinders complete cure and limits the success of anticancer drug therapy. For example, the differential expression of β-tubulin isoforms has been shown to result in resistance to anti-tubulin chemotherapeutics such as paclitaxel, along with increasing tumor aggressiveness 56,57. Another emerging form of taxane resistance is the increased expression of multidrug resitance (MDR) proteins – efflux pumps that remove cancer drugs from the tumor cell cytoplasm – such as the ATP Binding Cassette Subfamily B Member 1 (ABCB1 protein), P-glycoprotein and the Multidrug resistance-associated protein 1 (MRP1) 58,59.

The side effects of antimitotics are also related to the ubiquitous function of targets such as microtubules, which act in mitosis, interphase and quiescence. Drugs that disrupt microtubule function are associated with side effects such as neurotoxicity, which may lead to irreversible neuropathies. The current generation of antimitotic therapies in clinical trials did not meet the expectations of reduced side effects, even with diminished neurotoxicity 60. In fact, new side effects were found in patients treated with antimitotics and included hematological changes such as neutropenia 61-63. Next, we review the novel classes of antimitotics under preclinical investigation by our research group, which have the potential to overcome or minimize the limitations of current anticancer antimitotics. These molecules are synthetic analogs of well-known microtubule-targeting agents (combretastatins) and natural products with multiple or unknown targets (withanolides and pterocarpans).

### Combretastatins

The combretastatins are a potent class of natural products first isolated by Pettit and collaborators from the bark of the African bush willow, *Combretum caffrum* 64,65. These molecules are phenolic stilbenes that bind to the colchicine binding site on tubulin and lead to microtubule disruption and mitotic arrest by preventing spindle formation 66. Among the combretastatins, combretastatin A-4 (CA-4) is a lead compound that binds to the colchicine site and exerts antiproliferative activity by inhibiting tubulin polymerization. Despite its structural simplicity and potent activity, this natural product has problems related to its low solubility in natural biological systems and the lability of the *cis*-stilbene moiety. However, CA-4 is one of the most actively researched lead molecules in anticancer drug development programs worldwide, and extensive studies have been conducted to develop new CA-4 analogs to overcome the issues inherent to CA-4 and improve its pharmacotherapeutic profile 67.

Most of the recent structural modifications introduced in CA-4 have been based on changes in the *cis* olefinic bond, including the restriction of the *cis* configuration by a heterocyclic moiety and the replacement of this configuration by a stable linker. These modifications can maintain the original aromatic rings or can be associated with simultaneous alterations in either ring A or ring B 68. Among the new linkers explored to replace the *cis* olefinic bond of the CA-4 structure, N-acylhydrazone (NAH) is worth mentioning. From the synthetic and medicinal chemistry perspectives, NAH is considered a versatile subunit, and it has been described as a privileged structure 69. This review highlights some NAH derivatives that perturb microtubule/tubulin dynamics and have been synthesized by our research group using LASSBio^®^ compounds from the Federal University of Rio de Janeiro.

**Figure 2:**
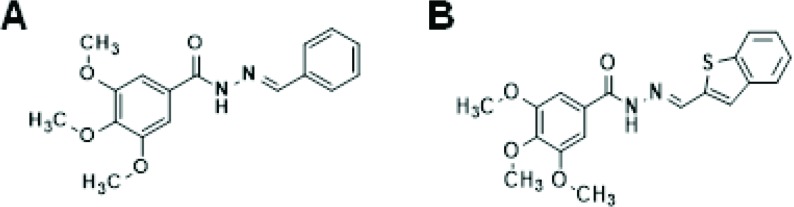
Combretastatin A-4 analogs. (A) LASSBio-1586. (B) LASSBio-1920.

Amaral et al. 70 identified a new series of CA-4 analogs designed in light of previous structure–activity relationship studies on CA-4 derivatives 71. Several compounds exhibit moderate to high antiproliferative activity in tumor cells, with IC_50_ values ranging between 18 and 4 nmol.L^-1^. Among these compounds, LASSBio-1586, patent number WO2013142935A1, has emerged as a lead antitumor candidate; LASSBio-1586 is structurally simple, able to inhibit microtubule polymerization, exhibits a broad *in vitro* and *in vivo* antiproliferative profile and has a better cytotoxic selectivity index than the natural prototype CA-4 70. In addition, the antiproliferative activity was evaluated in human lymphocytes to determine the selectivity indexes of the LASSBio derivatives, which were better than that of CA-4 72. More recently, our group described the synthesis of nine new isosteres of LASSBio-1586, aimed at improving the cytotoxicity and selectivity of LASSBio-1586 73. Among these derivatives, LASSBio-1920 was selected as the most potent, with IC_50_ values of 0.3, 9.0 and 5.0 nmol.L^-1^ in the HL-60, OVCAR-8 and HCT-8 tumor cell lines, respectively (Table 1). LASSBio-1920 also exhibited a notable cytotoxic selectivity index in comparison with that of CA-4 and LASSBio-1586 (Table 2, Figure 2), and its ability to modulate microtubule polymerization was confirmed. Docking studies indicated that LASSBio-1920 can interact with the same amino acid residues involved in the interaction of DAMA-colchicine as a cocrystal with β-tubulin.

Our data from structure–activity relationship studies on combretastatins reveal that the presence of the methoxy (OCH3) group linked at position 5 of the aromatic subunit (ring A) is crucial for cytotoxic activity, while the OCH3 group at position 4 is not. Concerning the aromatic rings linked to the imine subunit (ring B) of the isostere, our data suggest a direct relationship between the increase in lipophilicity and the enhancement of cytotoxic activity, in agreement with previous results of Amaral et al. 70. These findings can be explained on the basis of the improved hydrophobic interaction with β-tubulin 70. The more lipophilic the group bound to ring B is, the stronger the interaction with β-tubulin. However, the addition of lipophilic/hydrophobic groups to the combretastatin core should be carefully considered, since this structural modification may impair aqueous solubility and pharmacokinetic properties.

On the basis of the articles discussed here, we observed that microtubules and their associated proteins are feasible targets in drug discovery programs, particularly for the treatment of cancer. Numerous potent CA-4 derivatives have been developed in recent years, although very few have been accepted for clinical studies. Therefore, further structure-based drug discovery and optimization steps focusing on the CA-4 molecule and its analogs should be supported and encouraged to overcome the limitations of CA-4.

### Withanolides

Withanolides are lactones based on the cholesterol-derived ergostane molecule, with oxygen atoms enzymatically added to carbons of the steroidal core and its side chains, thus producing complex and unusual structures. These compounds are secondary metabolites found in plants of the family Solanaceae 74 and exhibit multiple biological activities, such as antitumor 75, cytotoxic 76, antistress 77, antifeedant 78, immunosuppressive 79 and antimicrobial 80 activities.

One of the most heavily investigated withanolides is withaferin A, which was originally isolated from the medicinal plant *Withania somnifera* (L.) Dunal, also called Ashwagandha or Indian ginseng. This molecule exhibits growth-inhibitory activity in many tumor cell lines through multiple mechanisms, including an antimitotic mode of action. Like taxanes and vinca alkaloids, withaferin A binds to β-tubulin, disorganizes the mitotic spindle and causes cell cycle arrest at the G2/M phase transition in breast cancer cells 81. This compound also impairs spindle assembly checkpoint function 82 and alters the expression levels of mitosis-related proteins, such as WEE1, phospho-histone H3, cyclin-dependent kinase inhibitor 1 (p21 protein), Aurora B 83 and the anaphase-promoting complex (APC) substrate securin 84. Withaferin A also targets a variety of nonmitotic proteins, such as vimentin 85, annexin II 86, heat shock protein 90 87 and the proteasome 88. Although these proteins function mainly during interphase, affecting them may prevent mitosis and inhibit cell cycle progression. The exact mechanism of action of withaferin A is not fully understood and may encompass different cell functions during different cell cycle phases.

Another group of withanolides called the withaphysalins was isolated from the plant *Acnistus arborescens*. Members of this group have chemical structures closely related to those of withaferin A and have also been studied by our research team 89-91. Rocha et al. showed that withaphysalin F and withaferin A may have mechanisms in common because the antiproliferative activity of withaphysalin F and its effect of arresting cells at the G2/M phase transition were also due to interference with microtubule polymerization 91.

### Pterocarpans

Pterocarpans are compounds extracted from the plant families Fabaceae, Leguminosae, Papilionaceae and Bituminaria and comprise the second largest group of isoflavonoids. Pterocarpans are phytoalexins produced by plants to defend against adversities such as resource shortages, pathogens and UV radiation 92. In ethnopharmacology, these compounds are extensively used to treat different ailments. These compounds have interesting biological activities, such as snake antivenom 93, anti-HIV 94, anti-*Trypanosoma* spp., anti-*Leishmania* spp. 95, antifungal, antibiotic 96, and topoisomerase I-inhibiting activities 97. Pterocarpans have a benzofuran–benzopyran tetracyclic ring system, which contains two chiral centers derived from the flavonoid skeleton at the 6a and 11a positions 96,98.

Hundreds of different pterocarpans have been discovered through different extraction methods, and the pharmacological activity of these pterocarpans has been tested. Since the basic structure of these compounds is chiral, methods to synthesize pterocarpans have been suggested, for example, sodium borohydride reduction of 2'-hydroxy isoflavones, Heck arylation of 2H-chromenes, Claisen rearrangement of aryl allyl ethers, aldol condensation between phenylacetates and benzaldehydes, 5-endo-trig radical cyclization, alkene metathesis and many others 98. (+)2,3,9-Trimethoxypterocarpan was isolated from *Platymiscium floribundum* in Brazil, and the convergent syntheses of its racemic and enantiomerically pure forms was developed by Dr. M. Banwell at the Australian National University (ANU). The synthetic route was patented in 2012 [WO2013000054 (A1)]. The molecule synthesized by Professor Banwell showed the same ^1^H NMR spectrum as the natural product 99.

**Figure 3:**
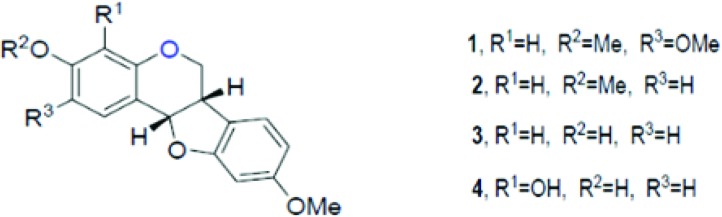
Structure of pterocarpans isolated from *Platymiscium floribundum* by Falcão et al. 95.

The compound (+)2,3,9-trimethoxypterocarpan has been investigated by our research group (Figure 3) as a potential anticancer compound 99. This molecule showed cytotoxicity against four leukemia cell lines (HL-60, Molt-4, Jurkat, and K562), and the proliferation of K562 cells was blocked after a 48-hour incubation period (at an IC_50_ of 0.8 µg.mL^-1^) 99,100. Flow cytometric analysis of HL-60 cells treated for 3 hours with (+)2,3,9-trimethoxypterocarpan showed the inhibition of DNA synthesis using a bromodeoxyuridine (BrdU) uptake assay. The pterocarpan inhibited BrdU incorporation by 37, 38 and 56% during the S phase at concentrations of 3.97, 7.95 and 15.9 μmol.L^-1^, respectively. After a 24-hour treatment with the same concentrations, propidium iodide-stained HL-60 cells showed no significant membrane damage but DNA fragmentation was seen in almost 50% of cells permeabilized with Triton X-100. Most HL-60 cells were arrested at the G2/M phase transition with tetraploid DNA content after treatment with 15.9 μmol.L^-1^ (+)2,3,9-trimethoxypterocarpan. In the indirect determination of mitochondrial depolarization via rhodamine 123, 38.6% of the HL-60 cell population exhibited depolarized mitochondria at the lowest treatment concentration. The results of the BrdU, membrane integrity and DNA content experiments suggested that (+)2,3,9-trimethoxypterocarpan is a cytostatic agent, while the results of the DNA fragmentation and mitochondrial depolarization experiments suggested that this pterocarpan induces apoptosis in HL-60 cells 101.

**Figure 4:**
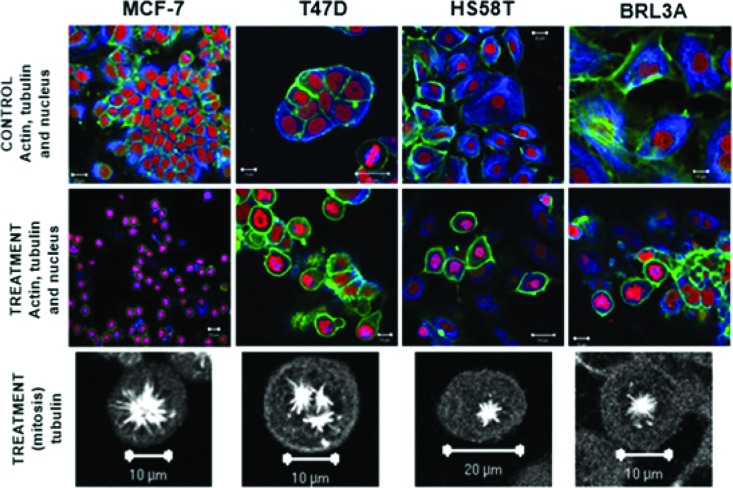
Formation of atypical mitotic spindles in 2,3,9-trimethoxypterocarpan-treated cells. Actin (green), tubulin (blue) and nuclei (red) are labeled in breast carcinoma and nontumor (BRL3A) cells treated or not treated with the compound. Note the normal arrangement of tubulin and actin fibers in the control cells; the formation of monopolar spindles in MCF-7, HS58T and BRL3A cells; and the formation of tripolar spindles in T47D cells. Figure from Militão et al. 97.

The cytostatic activity of (+)2,3,9-trimethoxypterocarpan in leukemia cells was corroborated by immunocytochemical experiments in the human breast adenocarcinoma cell lines MCF-7, T47D and HS58T. After treatment with 7.95 μmol.L^-1^ (+)2,3,9-trimethoxypterocarpan, the breast cancer cells remained arrested in mitosis with intact actin and microtubule networks. The same result was observed in nontumor BRL3A (murine liver) cells. However, the arrangement of microtubules was modified in most dividing cells, which exhibited monopolar mitotic spindles. Tripolar spindles were also observed, mainly in T47D cells (Figure 4). Such aberrant configurations prevented the alignment of chromosomes on the metaphase plate, blocking cell cycle progression and, consequently, cell division. Interestingly, none of the treated interphase cells exhibited microtubules with morphological alterations, suggesting that the effect of (+)2,3,9-trimethoxypterocarpan is restricted to mitosis. Lamin B labeling showed nuclear envelope disruption and confirmed cell cycle arrest at prometaphase 102. The monastral spindles observed in treated breast cancer cells resembled the phenotype induced by monastrol treatment 103,104. Monastrol impairs the acquisition of bipolarity in dividing cells without affecting centriole duplication 105, apparently due to its activity against the Eg5 kinesin 106.

## CONCLUSION

Antimitotic chemotherapeutics such as Taxol (paclitaxel), a microtubule-stabilizing agent that causes mitotic arrest, are currently successfully employed in cancer treatment 107. Regardless of the relative success of antimitotics, however, the death rate from cancer remains unacceptably high worldwide, and many efforts on different fronts have been made to improve cancer therapy 108. The reduction of drug resistance and side effects associated with antimitotics, such as neurotoxicity and bone marrow suppression, is a necessary improvement 60-63. In this review, we presented new antimitotic classes investigated by our research group that address the main issues of current chemotherapeutics.

The combretastatin derivatives are analogs of CA-4, an inhibitor of tubulin polymerization and microtubule formation 68. Because the functions of tubulin and microtubules are not limited to mitosis, CA-4 may affect quiescent or interphase cells, likely causing side effects in normal tissues 109. Thus, our structure–activity relationship studies identified structural characteristics of improved CA-4 analogs. We showed that the stability issues related to the *cis* stilbene nature of the combretastatin core were overcome by the replacement of the *cis* olefin double bond with an NAH framework, whose variation may result in inactive compounds. We discovered that compounds with the aromatic rings (A and B) in a *trans* configuration maintain tubulin-binding activity. Last, we showed a direct relationship between the increase in hydrophobicity and the cytotoxic effect of CA-4 analogs. Therefore, we described combretastatin analogs with increased cytotoxicity and selectivity toward tumor cells that can be lead molecules for new antimitotic agents inducing fewer side effects.

We also reviewed antimitotics derived from the natural products withanolides and pterocarpans. Indeed, natural sources are major sources of lead molecules and new drugs 110 such as CA-4 and Taxol. Although the mechanism of action of withanolides (withaphysalin F and withaferin A) is not fully understood, the targets of these agents may be proteins functioning in interphase, such as β-tubulin, vimentin, annexin II, heat shock protein 90 and proteasome subunits 81,85-88. Although these proteins are not mitosis-specific, their modulation may disrupt the cell cycle. Thus, withanolides may have greater-than-expected activity against targets of cancer cells in interphase 53,54, which could decrease the development of resistance to chemotherapy.

Finally, (+)2,3,9-trimethoxypterocarpan is an antimitotic agent that induces cell cycle arrest at the G2/M phase transition in leukemic cells and causes the aberrant organization of mitotic spindle microtubules in breast cancer cells. Although treatment of cells in M phase was found to result in monopolar and tripolar mitotic spindles, cells treated in interphase exhibited no microtubule modifications 102. These characteristics resemble the phenotype of cells treated with monastrol, a specific inhibitor of the kinesin motor protein Eg5, which is expressed only during mitosis 10,11. Such lines of evidence suggest that the target of (+)2,3,9-trimethoxypterocarpan is a mitosis-specific protein, and therefore, the action of this compound may be restricted to cells in mitosis. Therefore, this compound could potentially spare normal quiescent and interphase cells during clinical treatment, preventing neurotoxicity, for example.

All compounds reviewed demonstrate the potential to address important issues regarding current antimitotic-based cancer therapy. Therefore, *in vivo* antitumor assays will be performed to confirm these antimitotics as valuable lead molecules for the development of new anticancer drugs. However, specificity and toxicity issues concerning such molecules may arise but can be solved by combining antimitotics with other antineoplastic agents and/or monoclonal antibodies designed to bind to surface tumor antigens. This strategy can be implemented through direct covalent conjugation or drug encapsulation in nanoparticles functionalized with antibodies. Antibody-drug conjugates are a new trend in anticancer research 111; this approach also spurs the search for new and improved antimitotics.

## AUTHOR CONTRIBUTIONS

Paier CR conceptualized the study and edited and supervised the manuscript writing. Maranhão SS wrote the “Introduction”, “Antimitotic agents and their main targets” and “Challenges in the search for new mitotic targets for anticancer therapy” sections. Carneiro TR and Lima LM wrote the “Combretastatins” section. Rocha DD wrote the “Withanolides” section. Santos RS and Farias KM wrote the “Pterocarpans” section. Moraes-Filho MO and Pessoa Claudia revised the manuscript.

## Figures and Tables

**Table 1:** Cytotoxic activity (mean IC_50_±SEM) of LASSBio-1586 analogs and the CA-4 and LASSBio-1586 standards in tumor cell lines and peripheral blood mononuclear cells (PBMCs) after 72h of treatment (IC50 values are in µmol.L-1). SEM, standard error of the mean; IC50, the concentration of a compound needed to reduce cell growth by 50% *in vitro.*

Compound	HL60 (mean IC_50_±SEM)	OVCAR-8 (mean IC_50±_SEM)	HCT-8 (mean IC_50_±SEM)	PBMCs (mean IC_50±_SEM)
LASSBio-1837	0.047±1.0×10^-3^	0.099±0.010	0.27±0.020	0.24±0.045
LASSBio-1839	0.34±0.028	1.26±0.24	1.08±0.25	0.51±0.020
LASSBio-1840	0.10±0.031	0.39±0.031	0.34±0.068	0.43±8.1×10^-4^
LASSBio-1917	0.074±0.018	0.55±0.37	0.48±0.032	0.71±4.0×10^-3^
LASSBio-1918	0.75±0.085	4.26±0.20	4.18±0.17	2.14±0.61
LASSBio-1919	0.081±0.029	0.31±0.19	0.33±0.019	0.33±0.086
LASSBio-1920	3.0×10-4±4.0×10^-5^	9.0×10-3±6.0×10^-3^	5.0×10-3±3.0×10^-3^	0.10±0.011
CA-4	2.2×10-3±3.0×10^-4^	5.0×10-4±1.0×10^-4^	5.0×10-3±1.0×10^-3^	3.2×10^-3^
LASSBio-1586	0.29±1.2×10^-3^	0.29±0.040	0.45±0.081	1.34±0.065

**Table 2:** Cytotoxic selectivity index (SI), which represents the IC_50_ for PBMCs/cancer cell lines after 72h of treatment. PBMCs, peripheral blood mononuclear cells; SEM, standard error of the mean; IC50, the concentration of a compound needed to reduce cell growth by 50% *in vitro.*

Compound	SI PBMC/HL-60	SI PBMC/OVCAR-8	SI PBMC/HCT-8
LASSBio-1920	333.30	11.11	20.0
LASSBio-1586	4.60	4.60	3.0
CA-4	1.45	6.40	0.60
